# Antifungal and Reactive Oxygen Species Response of *Saccharomyces cerevisiae* to Deep Eutectic Solvents for Bacterial Nanocellulose-Based Eutectogel

**DOI:** 10.1021/acsphyschemau.6c00057

**Published:** 2026-06-25

**Authors:** Maurelio Cabo, Kwaniyah Tuffour, Dennis LaJeunesse

**Affiliations:** Department of Nanoscience, Joint School of Nanoscience and Nanoengineering, University of North Carolina Greensboro, Greensboro, North Carolina 27455, United States

**Keywords:** antifungal, deep eutectic solvents, bacterial nanocellulose, eutectogels, antibiotic, fungi

## Abstract

Antifungal resistance is an emerging global challenge that requires new antimicrobial materials and mechanistic insights. This study aims to evaluate the antifungal activity and reactive oxygen species (ROS) response of *Saccharomyces cerevisiae* (TBR1) exposed to imidazole-based deep eutectic solvents (DESs) and ternary DESs (TDESs), including their incorporation into bacterial nanocellulose (BNC) eutectogels. Minimum inhibitory concentration (MIC) results show concentration-dependent inhibition, with DES exhibiting higher antifungal potency than TDES. However, TDES induces significantly greater ROS production, indicating distinct mechanisms of action. Dead cell assay and scanning electron microscopy (SEM) imaging reveal increased cell death and morphological changes without membrane rupture, supporting oxidative stress-driven intracellular damage. Proton nuclear magnetic resonance (^1^H NMR) metabolite analysis indicates disruption of carbohydrate metabolism and energy homeostasis. Disk diffusion confirms that antifungal activity is primarily driven by imidazole and retained in eutectogel systems, although with reduced diffusion. Herein, DES and TDES act through membrane interaction, metabolic disruption, and ROS-mediated damage, while BNC integration enables sustained antimicrobial delivery for potential biomedical, drug delivery system, and wound dressing applications.

## Graphical abstract

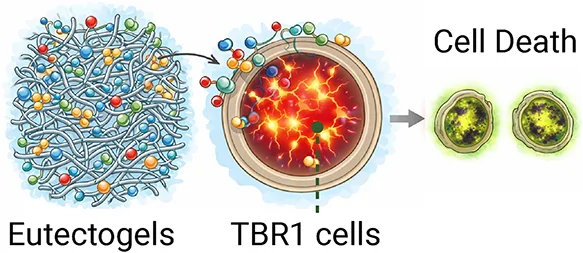

## 1 Introduction

Antimicrobial resistance has become a major global health concern, affecting not only bacterial pathogens but also fungal organisms that are increasingly difficult to treat.^1^ In recent decades, the incidence of antifungal-resistant infections has risen steadily, leading to significant challenges in clinical management.^2^ Although fungal infections occur less frequently than bacterial or viral infections, they often result in severe and life-threatening complications, particularly in immunocompromised individuals such as patients with HIV/AIDS, those undergoing chemotherapy, transplant recipients, and premature infants.^3^ The growing number of resistant fungal strains, combined with the limited availability of effective antifungal agents, highlights the urgent need to explore new strategies for controlling fungal growth and understanding the cellular responses involved in the antifungal activity.

Compared with bacterial and viral diseases, fungal infections remain relatively understudied, despite their increasing clinical importance. One of the reasons for this gap is the complexity of fungal cells, which are eukaryotic and share many similarities with human cells, making it difficult to achieve selective toxicity. As a result, antifungal therapies often show limited specificity and may produce adverse side effects. Over the past century, the number of fungal infection cases has increased dramatically, especially in developed countries, where the population of immunocompromised patients has grown due to advances in medical treatments that prolong life. Epidemiological studies have reported a significant rise in fungal-related sepsis, with the number of cases in the United States increasing by more than 200% between 1979 and 2000.^3^ These observations emphasize the importance of investigating fungal physiology, stress responses, and potential antifungal agents that can be used in biomedical and materials science applications.

Among fungal species, *Saccharomyces cerevisiae* has attracted considerable attention due to its dual role as both a beneficial microorganism and a potential opportunistic pathogen.^3^ This yeast is widely distributed in nature and is best known for its use in baking, brewing, and fermentation industries, where it has been used for thousands of years. In addition, certain strains have been used as probiotics and nutritional supplements, such as *S. boulardii*, which is commonly employed in the treatment of antibiotic-associated diarrhea. Because of its long history of safe use, *S. cerevisiae* has traditionally been regarded as a harmless, nonpathogenic organism. However, clinical reports over the past few decades have demonstrated that this yeast can cause infections under specific conditions, particularly in individuals with weakened immune systems.^4^ Cases of *S. cerevisiae* infection have been associated with a wide range of clinical manifestations, including vaginitis, cutaneous infections, bloodstream infections, and systemic involvement of vital organs. These findings have led to the reclassification of *S. cerevisiae* as an emerging opportunistic pathogen, especially in hospital environments, where immunosuppressed patients are more vulnerable.

The increasing recognition of *S. cerevisiae* as a potential pathogen has stimulated interest in understanding its stress-tolerance mechanisms and cellular defense responses. Many antifungal agents exert their activity by inducing oxidative stress, thereby disrupting the cellular homeostasis. Therefore, evaluating the minimum inhibitory and ROS responses of *S. cerevisiae* provides valuable insights into the antifungal potential of new chemical compounds and materials.

In recent years, DESs have emerged as a new class of green and tunable solvents with promising applications in biotechnology, medicine, and materials science.^5^ Traditional eutectic solvents and DESs should be clearly differentiated, while all eutectic mixtures exhibit melting point depression, DESs are specifically characterized by strong intermolecular interactions, typically hydrogen bonding between hydrogen-bond donors (HBDs) and hydrogen-bond acceptors (HBAs), which generate significant negative deviations from thermodynamic ideality and lead to a much deeper melting point depression than expected for ideal mixtures. Mercadal et al. emphasized that DESs arise from stronger heteromolecular interactions than those present in the pure components and noted that some systems commonly classified as DESs may behave as conventional eutectic solvents.^6^ Abranches and Coutinho further stressed that melting point depression alone is insufficient to classify a system as a DES, since all eutectic mixtures inherently display cryoscopic depression.^7^ They proposed that DESs should be defined thermodynamically as eutectic solvents exhibiting enthalpy-driven negative deviations from ideality because of favorable interactions between the mixture components.

These solvents are attractive because they are easy to prepare, cost-effective, biodegradable, and often less toxic than conventional organic solvents.^8^ In addition to their role as solvents, DESs have shown biological activity, including antimicrobial and antifungal effects.^9^ However, the mechanisms by which DESs interact with fungal cells and induce cellular stress responses remain insufficiently understood. Investigating how DESs affect yeast growth and oxidative stress pathways can provide important information about the design of functional biomaterials with antimicrobial properties. Herein, we explore our recently studied and developed imidazole-based DES systems composed of choline chloride/imidazole, a binary DES, and choline chloride/imidazole/tannic acid, a ternary DES.^5,8^

Parallel to the development of new antimicrobial agents, hydrogels have gained significant attention as advanced materials for biomedical applications, particularly in wound care.^10^ Hydrogels are three-dimensional networks of hydrophilic polymers capable of absorbing large amounts of water while maintaining the structural integrity. Their high water content, softness, and permeability make them suitable for use as wound dressings, where they can provide a moist environment that promotes tissue regeneration and allows oxygen exchange. Moreover, hydrogels can be used as carriers for antimicrobial compounds, enabling localized delivery directly at the wound site.^11^

This approach reduces systemic exposure to drugs, minimizes toxicity, and helps preserve normal microbiota. Despite these advantages, many conventional antimicrobial hydrogels rely on passive release mechanisms, which often lead to uncontrolled drug delivery and reduced therapeutic efficiency. Such limitations may contribute to the development of resistant microorganisms and decrease the effectiveness of the treatment.

To overcome these challenges, researchers have focused on the development of stimuli-responsive or “smart” hydrogels that can interact with the biological environment and release active compounds only when needed.^12^ These systems offer improved control over drug delivery and enhance antimicrobial performance. Among the different types of hydrogels, bacterial nanocellulose-based hydrogels have attracted special interest because of their excellent mechanical strength, biocompatibility, and high water-holding capacity.^13,14^ BNC is a natural polymer produced by certain microorganisms and is known for its purity, high crystallinity, and ability to form stable three-dimensional networks.^15^ When combined with deep eutectic solvents, these materials can form eutectogels, which integrate the structural advantages of nanocellulose with the functional properties of DESs. Such eutectogels have the potential to act as antimicrobial materials with controlled activity, making them promising candidates for wound dressing and biomedical applications.^15–17^

However, before these materials can be safely applied, it is necessary to evaluate their biological effects at the cellular level, particularly their antifungal activity and their ability to induce oxidative stress. *Saccharomyces cerevisiae* serves as an ideal model organism for this purpose because of its well-known physiology, ease of cultivation, and relevance to opportunistic fungal infections. By analyzing the growth inhibition and ROS response of *S. cerevisiae* when exposed to deep eutectic solvents, valuable information can be obtained regarding the safety and effectiveness of the DES-based eutectogels. Understanding these interactions will contribute to the rational design of antimicrobial biomaterials that are effective against pathogens and compatible with biological systems.

Therefore, this study aims to investigate the antifungal activity and ROS response of *Saccharomyces cerevisiae* exposed to DES intended for use in bacterial nanocellulose-based eutectogels. The findings of this research are expected to provide insight into the cellular mechanisms involved in DES-induced stress and support the development of advanced antimicrobial hydrogel systems for biomedical applications, particularly in infection control and wound healing.

## 2 Experimental Section

### 2.1 Materials

Fisher Scientific (Thermo Fisher Scientific, Waltham, MA) provided yeast, peptone, dextrose, 2xYT Agar, tannic acid, and choline chloride (99%). The fluorescent dye 2′,7′-dichlorodihydrofluorescein diacetate (H2 DCFDA) and imidazole (99%) were acquired from Sigma-Aldrich (St. Louis, MO). No purification was performed on the compounds utilized in this investigation; they have been used in their original form. The wild-type TBR1 FlO11 *Saccharomyces cerevisiae* cell strain was employed as a bioactivity model. In Table S1, the sample weight and the final concentration of each component and the DES system are disclosed.

### 2.2 Synthesis of DES and TDES

The deep eutectic solvent (DES) was synthesized by combining choline chloride (8.39 g) and imidazole (9.53 g) at a 3:7 molar ratio, determined from prior eutectic phase investigations indicating stable liquid formation at around 70 mol % imidazole composition.^18^ Hou et al. conducted a systematic evaluation of the melting behavior of ChCl/imidazole mixtures across different compositions, revealing that mixtures with approximately 70 mol % imidazole displayed eutectic properties and markedly reduced melting temperatures compared to the pure components.^18^ The ChCl–Imi system at a 3:7 molar ratio produces a stable homogeneous liquid phase with reduced melting characteristics due to significant hydrogen-bond interactions between chloride anions and the N–H group of imidazole.

In our recently published investigation, the combination was subjected to heating at a high temperature of 120 °C on an analog hot plate for 3 h, which included about 1 h for preheating and 1 h for postheating in order to adjust for gradual temperature stabilization.^5^ Upon heating, the mixture converted into a uniform yellowish liquid, indicating the formation of a deep eutectic solvent (DES). The obtained liquid was allowed to stabilize at ambient temperature for 24 h to confirm its liquid stability. Subsequently, tannic acid (0.5% w/v in relation to the total volume of the deep eutectic solvent) was added to produce the ternary deep eutectic solvent (TDES). The ternary deep eutectic solvent (TDES) formulation used in this investigation was derived from our prior research on tailoring the semiconductive and electrochemical characteristics of ChCl–imidazole-based eutectic systems.^5^ Upon addition, the solution’s hue changed from yellowish to reddish-black, indicating more pronounced intermolecular interactions within the hydrogen-bond network.^5,8^

### 2.3 Fabrication of Never-Dried BNC and Eutectogels

*Gluconacetobacter hansenii* was cultivated in Hestrin–Schramm (HS) medium, which consisted of 2% (w/v) dextrose, 0.5% (w/v) yeast extract, 0.5% (w/v) peptone, 0.27% (w/v) Na_2_HPO_4_, and 0.125% (w/v) citric acid. An agar plate (85 mm × 15 mm) with identical HS medium composition and 2% agar was prepared for the inoculation of the bacterial strain and incubated at 30 °C for 48 h. A bacterial colony of pea size was then transferred from the inoculation plate to a 6-well plate (Corning Costar 6-well Clear TC-treated Multiple Well Plates) and cultivated for 7 days at 25 °C. Subsequent to the incubation time, the pellicles were subjected to a 0.1 M NaOH concentration at 95 °C for 1 h to eliminate bacterial cells and biofilm constituents, therefore isolating the bacterial nanocellulose hydrogels. The hydrogels were then rinsed with distilled water until achieving a neutral pH and were kept in deionized (DI) water at ambient temperature. Three mL of eutectic solvent was dispensed into each well of a 6-well plate, and then one hydrogel specimen was placed into each well. The plate was covered with foil and put in the fume hood for a duration of 3 days. After 3 days, the pellicles were detached and placed in a sterile Petri dish. The Petri dish was covered with foil with holes, and the excess eutectic solvent was allowed to evaporate gradually over a period of 7 days.

### 2.4 MIC

The microdilution assay was used to ascertain the minimum inhibitory concentration (MIC) of DES by evaluating the observable growth of microorganism in broth medium. A wild-type strain of TBR1 FlO11 *Saccharomyces cerevisiae* was cultured in Yeast-Peptone-Dextrose (YPD) medium at 30 °C in a 50 mL conical flask, agitated at 150 rpm for 48 h prior to sample processing. Cultures were grown to the mid logarithmic phase at an optical density of 0.5 at 600 nm (OD_600_). OD_600_ measurements were performed using a Thermo Scientific Nanodrop 2000C spectrophotometer. YPD medium (100 μL) was added to a 96-well round-bottom polystyrene microtiter plate (Costar, Corning Inc., Kennebunk), followed by a series of 2-fold dilutions of eutectic solvents. Subsequently, an inoculum of 10 μL was introduced to each well, concluding with additional YPD media to reach a final volume of 200 μL. The YPD medium containing inoculum but without eutectic solvents functioned as a control. Fungal growth in YPD medium supplemented with eutectic solvents was performed at 37 °C with agitation at 150 rpm for 24 h. Absorbance measurements (OD_600_) were acquired with the BioTek Cytation 5 Imaging Multi-Mode Reader. Experiments were performed in triplicate to ensure validity.

### 2.5 ROS Assay

A ROS experiment was performed to evaluate intracellular oxidation levels after the exposure of TBR1 cells to eutectic solvent treatments. TBR1 cells were cultured individually in 10 mL of YPD media. Experiments started when the cell culture reached an optical density (OD600) of 0.5. Fungal cell pellets from the culture medium were produced using centrifugation at 3500 rpm for 10 min. The cell pellets were washed with 1× Phosphate Buffer Saline (PBS), centrifuged again, and then resuspended in 1× PBS. Thereafter, cell solutions underwent sonication to lyse the cells and were then stained with 2 μL (1:2000 dilution) of 10 mM 2′,7′-dichlorodihydrofluorescein diacetate (H2 DCFDA) fluorescent dye. The cells were then incubated at 37 °C with agitation for 30 min. Centrifugation was used to pellet the incubated cells, which were then rinsed with 1× PBS to remove remaining H2 DCFDA. Ten microliters of DES and TDES treatment were added to the cell solution, along with 200 μL of buffer in a 96-well plate, and incubated at 37 °C for 2 h. A 2-h time point was selected to reduce confounding variables from late-stage growth inhibition and cell death that might affect fluorescence-based ROS measurements. Untreated TBR1 cells served as the control sample. The fluorescence intensity for reactive oxygen species detection was measured using the BioTek Cytation 5 Imaging Multi-Mode Reader, using an excitation wavelength of 485 nm and an emission wavelength of 535 nm. The trials were performed in triplicate to ensure validity.

### 2.6 Dead Cell Assay

Dead cell viability in TBR1 *S. cerevisiae* was determined by using propidium iodide (PI) staining.^19^ A fresh PI staining solution was prepared by diluting the PI stock in PBS to the required final concentration, which was optimized empirically for yeast cells. Cultured cells were collected, washed once with PBS to remove residual medium, and resuspended in the staining solution to ensure complete coverage of the cell suspension. The cells were incubated for 5 min at room temperature in the dark to allow PI to penetrate membrane-compromised cells. Following incubation, the staining solution was removed by washing with PBS, and the cells were resuspended in PBS for the observation. Samples were immediately analyzed by using fluorescence microscopy to distinguish dead cells based on PI uptake. The staining solution was prepared fresh and used within 2 h to ensure consistent results.

### 2.7 SEM Morphology

The fungal samples for SEM imaging with a JEOL JSM-IT800 FESEM were prepared with a modified fixation approach derived from normal protocols. A combination of glutaraldehyde and formaldehyde (Karnovsky’s fixative) was used for primary fixation to maintain cellular morphology, mixed with 1x phosphate-buffered saline (PBS). The cell pellet samples were obtained after MIC and ROS assessments. Following the removal of the supernatant, 200 μL of a prepared stock solution of glutaraldehyde-formaldehyde/phosphate-buffered saline (PBS) combination was added to the cell pellet, centrifuged, and allowed to rest for 30 min to eliminate excess fixative and eutectic solvents. The fungal cells were then centrifuged and rinsed five times with autoclaved deionized water. Centrifugation was used throughout the rinse and dehydration phases to guarantee adequate pelleting of fungal cells. Low-speed centrifugation (3500 RCF for 10 min at ambient temperature) was used to prevent any harm to the fungal cells. A sequential dehydration process was conducted with escalating quantities of ethanol (25%, 50%, 75%, 95%, and 100%). After the dehydration process, a 10 μL cell culture was applied onto a clean silicon wafer for additional imaging analysis, and allowed to dry at room temperature for 24 h.

### 2.8 ^1^H NMR

The apparatus used for spectral acquisition was an Agilent 400 MHz, outfitted with a TB_MR1107W018 probe. The spectra were processed using MestReNova version 15.0.1 (Mestrelab Research, Santiago de Compostela, Spain). All trials were conducted at 25 °C. ^1^H NMR spectra were obtained within the range of −2 ppm to 14 ppm, using a d1 of 2 and 64 scans (ns), resulting in a total acquisition duration of 3 min and 30 s. All samples were prepared using deuterium oxide (D2O) as the solvent. Samples were collected post-MIC and ROS tests, followed by centrifugation to remove cells from the media/solvent used. For cell pellet samples, 200 μL of D2O solvent was introduced to an Eppendorf tube (600 μL), followed by vortexing, and then transferred to NMR tubes to combine with 400 μL of D2O solvent. For liquid control samples, 100 μL was combined with 500 μL of D2O solvent in an NMR tube after vortexing.

### 2.9 Plate-Hole Diffusion Assay

The 2 × YT agar was formulated in deionized water following the laboratory protocols established by Williams et al.^20^ The produced agar underwent sterilization by autoclaving at 121 °C for 15 min. After sterilization, the molten agar was allowed to cool to ambient temperature and was then aseptically dispensed onto sterile Petri plates. The plates remain undisturbed until completely hardened and sufficiently dried. A wild-type strain of TBR1 suspension was produced. A disposable plastic inoculum loop was used to uniformly distribute the fungal culture throughout the whole surface of the YPD agar plate, resulting in a consistent lawn of growth. The infected plates were allowed to equilibrate momentarily at room temperature to facilitate the absorption of surplus surface moisture. A sterile 7 mm diameter punch is used to form wells in the agar according to the methodology established by Hayhoe et al.^21^ The agar plugs are meticulously extracted and disposed of, and the wells are appropriately labeled. 100 μL of sample volume is dispensed into each well. The plates were thereafter maintained at room temperature for a prediffusion interval to facilitate the diffusion of compounds into the agar matrix. After diffusion, the plates are incubated at 30 °C for a duration of 24 h. Following incubation, antimicrobial efficacy was evaluated by measuring the diameter of inhibition zones surrounding the wells from the plate’s underside, utilizing the zone diameter interpretative standards established by Hudzicki, also referred to as the Kirby-Bauer disk diffusion susceptibility test protocol.^22^

### 2.10 Disk Diffusion Assay

Antimicrobial activity of the deep eutectic solvent-impregnated BNC hydrogel (eutectogel) against TBR1 cells was evaluated using an agar diffusion assay.^21^ YPD agar plates were prepared at uniform thickness and allowed to solidify before use. A fresh culture of TBR1 was suspended and evenly spread over the agar surface using a sterile swab to form a uniform lawn. Circular samples of the eutectogel containing the deep eutectic solvent were then placed on the inoculated agar surface. Never-dried BNC was used as a control. Plates were incubated at 30 °C over a 24 h period under growth conditions to allow cell growth and diffusion of the solvent from the hydrogel matrix into the surrounding agar. After incubation, antifungal activity was determined by observing and measuring the clear zones of inhibition around the eutectogel samples, indicating suppression of TBR1 growth.

### 2.11 Characterization of BNC-Based Eutectogels

The surface details of wet BNC and BNC-based eutectogels were measured using an Asylum-3D Origin + AFM in a noncontact way with Tap-150G AFM probes from Ted Pella, which have specific sizes and strengths. We conducted the measurements at ambient temperatures and processed the images using the Gwyddion software packages. The thermal analysis (TGA and DTG) was characterized using a thermogravimetric analyzer (TA Instruments TGA 5500) within a temperature range of 30 to 600 °C at a rate of 20 °C/min under an air atmosphere. To measure shear stress, viscosity, and normal stress, we used TA HR20 Rheometer equipment set at 25 to 45 °C.

## 3 Results and Discussion

### 3.1 Bioactivity Assay Analysis

Determining the MIC is an essential first step in evaluating the antifungal potential of any compound. MIC defines the lowest concentration of a substance that prevents visible microbial growth after a defined incubation period. The results presented in Figure 1 (a–d) illustrate the response of *TBR1* cells when exposed to DES and TDES, evaluated through optical density (OD_600_) and percentage cell viability.

**Figure 1:**
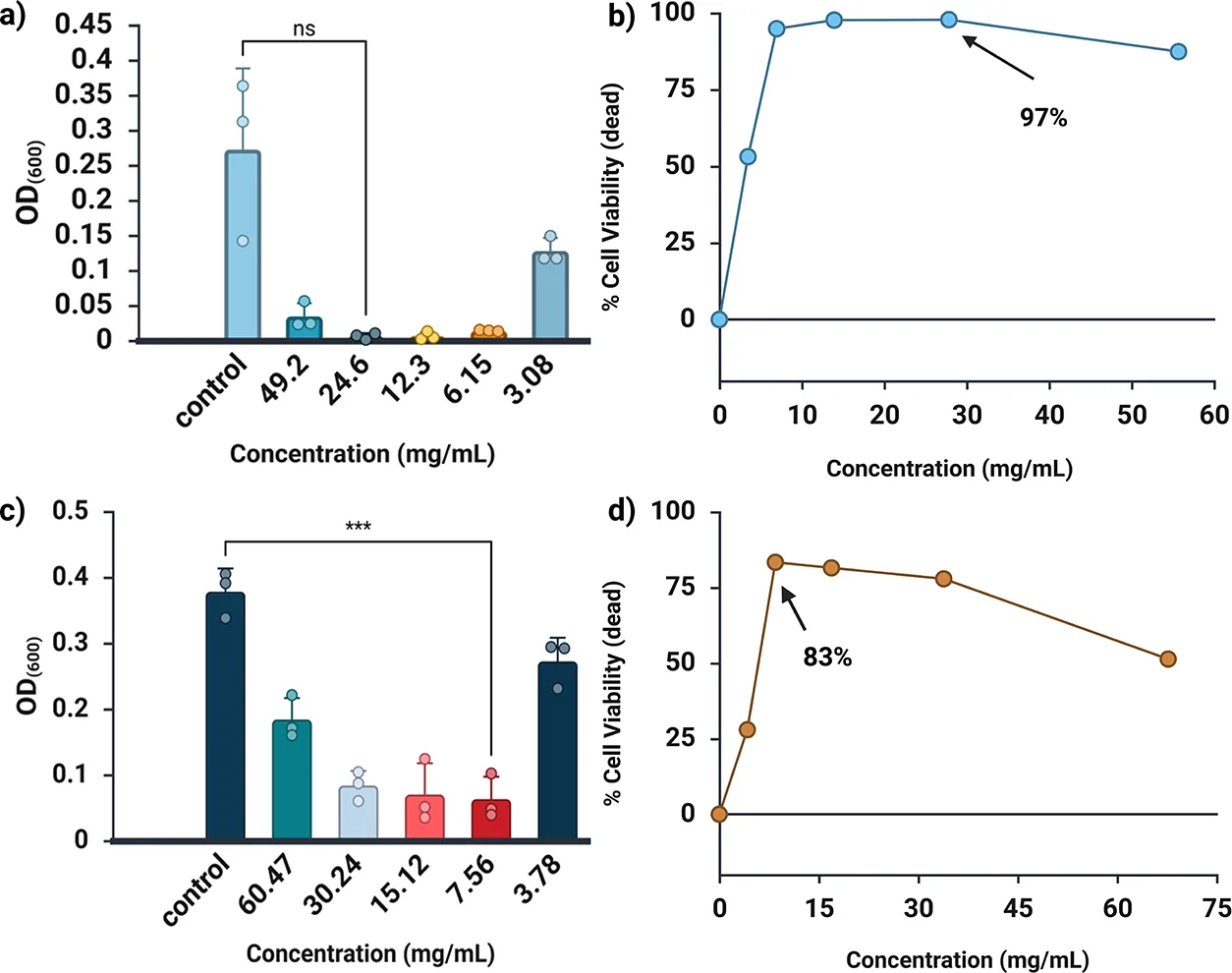
The antifungal response of TBR1 cells following exposure to DES (a) and TDES (c) across a range of concentrations. Growth inhibition was evaluated using optical density (OD_600_), while cell viability (b, d) was assessed as percentage cell death. The significance compared to the untreated fungal cells was determined using ANOVA one-way analysis followed by *t* test, ns = *P* = not significant, and *** = *P* < 0.001.

For DES exposure (Figure 1(a)), optical density (OD_600_) decreases as concentration increases from 3.08 to 24.60 mg/mL, indicating reduced cell growth. This trend is consistent with the expected behavior near the MIC, where increasing concentrations begin to inhibit proliferation. The lowest OD values occur around 6.15–24.60 mg/mL, suggesting that these concentrations are close to or above the MIC. Interestingly, at the highest concentration, 49.20 mg/mL, OD increases again. This nonlinear response may reflect experimental variation or changes in solvent–cell interactions at higher concentrations, and it highlights that OD alone may not fully represent cell viability under all conditions. Figure 1(b) provides complementary insight through cell viability measurements. A sharp increase in cell death is observed with increasing DES concentration, reaching approximately 97% at around 24.60 mg/mL. The slight decrease in cell death at higher concentrations suggests that increasing dosage beyond the MIC does not necessarily enhance antifungal efficiency proportionally.^23^

For TDES exposure (Figure 1(c)), OD_600_ also decreases with increasing concentration, but the reduction is less pronounced compared to DES. Growth inhibition is evident; however, some residual growth persists even at higher concentrations, indicating that TBR1 cells exhibit slight partial tolerance to TDES. This observation is further supported by Figure 1(d), where the maximum cell death reaches approximately 83%, which is lower than that observed for DES. The highest antifungal effect occurs around 7.56 mg/mL, again consistent with MIC-based expectations, but the difference was statistically significant (*p* < 0.001). At higher concentrations, the effect slightly declines, possibly because of changes in the physical or chemical behavior of TDES that influence its interaction with cells.

Herein, results suggest that both DES and TDES exhibit concentration-dependent activity against TBR1 cells, with DES showing stronger inhibition and higher cell death, emphasizing the importance of optimizing concentration rather than simply increasing dosage.

The ROS experiment results shown in Figure 2a unveil a clear increase in oxidative stress in *TBR1* cells following exposure to the DES and TDES antifungal solvents. Illustrating endogenous ROS production under standard physiological conditions, the control condition (*TBR1*) exhibits a fluorescence intensity of 32,223.58 au. When exposed to DES, ROS levels increase to 42,435.75 au, revealing that DES induces oxidative stress beyond *TBR1*’s normal levels. TDES ignites an even more pronounced effect, as its exposure leads to more than double the fluorescence intensity of the *TBR1* control (76,6686.75 a.u). Though both solvents stimulate ROS generation, TDES triggers a significantly stronger oxidative response in *TBR1* cells.

**Figure 2:**
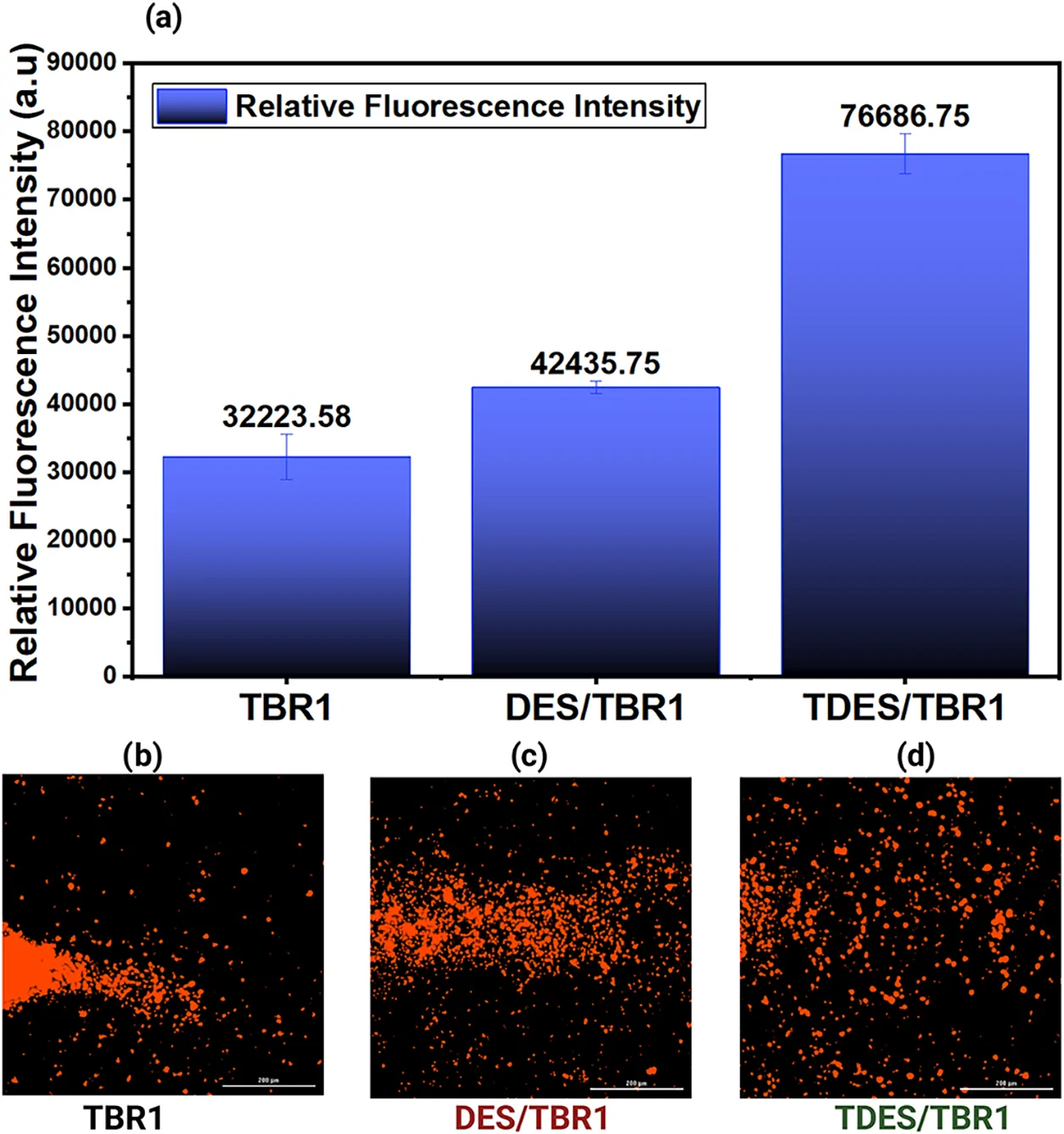
(a) ROS response of TBR1 cells upon before and after exposure to deep eutectic solvents. (b–d) Dead cell analysis when treated with PI staining.

Heightened ROS levels observed under antifungal treatment can be correlated with cellular stress mechanisms in yeast. ROS in *S. cerevisiae* typically derives from mitochondrial respiration. When leaked electrons from the electron transport chain react with molecular oxygen, the oxygen is prematurely reduced from O_2_ to O_2_^–^.^24,25^ Elevated ROS levels can damage cell viability by impairing cellular structure, components, and proteins. Although yeast cells employ antioxidant defense systems to combat ROS, the ROS production can exceed the capacity of such systems. Acute oxidative damage may trigger apoptosis-like cell death.^26^

These ROS findings closely align with the MIC experimental results. As increasing concentrations of DES correspond to decreasing OD600, this strong antifungal effect is consistent with *TBR1’*s moderate DES-induced ROS increase. This correlation suggests that oxidative stress, because of DES, contributes to both growth inhibition and cell death, but may not be the only mechanism driving toxicity. The extent of cell death observed insinuates the involvement of additional cytotoxic mechanisms.

In contrast, TDES displays a distinct pattern. While the MIC data uncovered that TDES achieves a lower maximum cell death compared to DES, the ROS experiment highlights remarkably higher oxidative stress. Therefore, TDES may deploy its antifungal effects primarily through ROS-related mechanisms, rather than direct cytotoxicity alone. It is possible that activation of stress-response pathways such as the High-Osmolarity Glycerol (HOG) or the Cell Wall Integrity (CWI) pathway may have mitigated TDES-induced oxidative damage, aiding the persistence of some cell growth at higher TDES concentrations. The discrepancy between TDES-induced ROS levels and overall cell death emphasizes that ROS generation and antifungal efficacy do not always correlate linearly. Though TDES causes higher oxidative stress, DES proves to better promote complete cell death, suggesting additional supporting mechanisms. Overall, when integrated with MIC results, the data implies that ROS plays a critical but not exclusive role in TBR1’s antifungal activity. Optimal antifungal efficacy is dependent on oxidative damage combined with other disruptions to the cell.

The dead cell assay, in Figure 2(b,c,d) and Table 1, shows a clear increase in dead cell count following exposure to DES and TDES compared to the untreated TBR1 control. While the control sample (Figure 2(b)) exhibits relatively low cell death (330 cells), DES treatment (Figure 2(c)) results in a notable increase (901 cells) and TDES shows (Figure 2(d)) a substantially higher number of dead cells (2524 cells). This trend indicates that both DES and TDES induce cytotoxic effects in TBR1 cells, with TDES exhibiting a stronger impact in terms of total cell death. In addition to cell count, the average cell size decreases progressively from control (12.90 μm) to DES (10.66 μm) and further in TDES (8.8 μm). This reduction in cell size suggests cellular shrinkage, which is commonly associated with stress responses such as oxidative damage and apoptosis-like processes in *Saccharomyces cerevisiae*.^27^

**Table 1:** Dead Cell Assay Result

samples	cell count	ave. cell size (μm)
TBR1	330	12.90
DES/TBR1	901	10.66
TDES/TBR1	2524	8.8

When connected to the MIC results, the increased cell death aligns with concentrations that inhibit fungal growth, confirming that both DES and TDES are effective at suppressing proliferation. Furthermore, the ROS assay supports this interpretation as elevated ROS levels likely contribute to intracellular damage, leading to cell death.

A critical aspect of utilizing deep eutectic systems in aqueous biological assays is understanding their structural state upon dilution within the culture medium. While bulk eutectic properties change as water content increases, literature confirms that localized hydrogen-bonded supramolecular clusters between HBAs and HBDs remain structurally organized even under diluted conditions. To confirm the added value of the eutectic architecture over a simple diluted mixture of constituents, as shown in Table S2, we evaluated and showed the percent viability results from the MIC assay from individual components and the DES system. The standalone components exhibited baseline or vastly inferior antifungal efficacy when tested in isolation, see Figures S1 to S3. In contrast, the cohesive DES matrix drove complete fungal inhibition and profound intracellular ROS generation, see Figure S4. This clear, synergistic enhancement verifies that preassembling these elements into a eutectic system fundamentally alters their biological interaction dynamics, facilitating superior penetration or disruption of microbial defenses that simple solutions of individual constituents cannot achieve.

For the lower antifungal activity of the tannic-acid-containing TDES, this may result from TA altering the eutectic network rather than simply adding another antifungal component. The MIC data show that the individual components behave differently from the assembled DES systems: ChCl (Figure S1) and TA (Figure S3) alone produced strong cell growth across the tested range, whereas imidazole (Figure S2) showed high viability at higher concentrations but stronger cell growth at lower concentrations.

This suggests that antifungal activity depends on the supramolecular organization of the DES, not only on the intrinsic activity of each component. Incorporating TA likely increases hydrogen-bonding density and molecular structuring within the ternary system, which may reduce component mobility, alter diffusion into the fungal cell wall, and weaken membrane-disruptive interactions.^28^ In addition, TA’s polyphenolic structure may buffer ROS-mediated stress, consistent with the ROS response analysis (Figure S4). Thus, TA may partially antagonize the binary DES by stabilizing the eutectic matrix and reducing its interaction with fungal cells.

### 3.2 Cell Morphological Analysis

The SEM images (Figure 3) provide important visual support for the MIC and ROS assay results by illustrating how TBR1 (*Saccharomyces cerevisiae*) cells respond structurally when exposed to DESs and TDESs. In the control images (Figure 3(a,d)), the cells exhibit a smooth, round, and intact morphology, which is characteristic of healthy yeast cells. There are no visible surface irregularities or distortions, indicating that the cell walls and membranes remain stable. This observation is consistent with the MIC and ROS assay results, where untreated cells show normal growth and minimal cell death, confirming that the baseline conditions do not induce stress.

**Figure 3:**
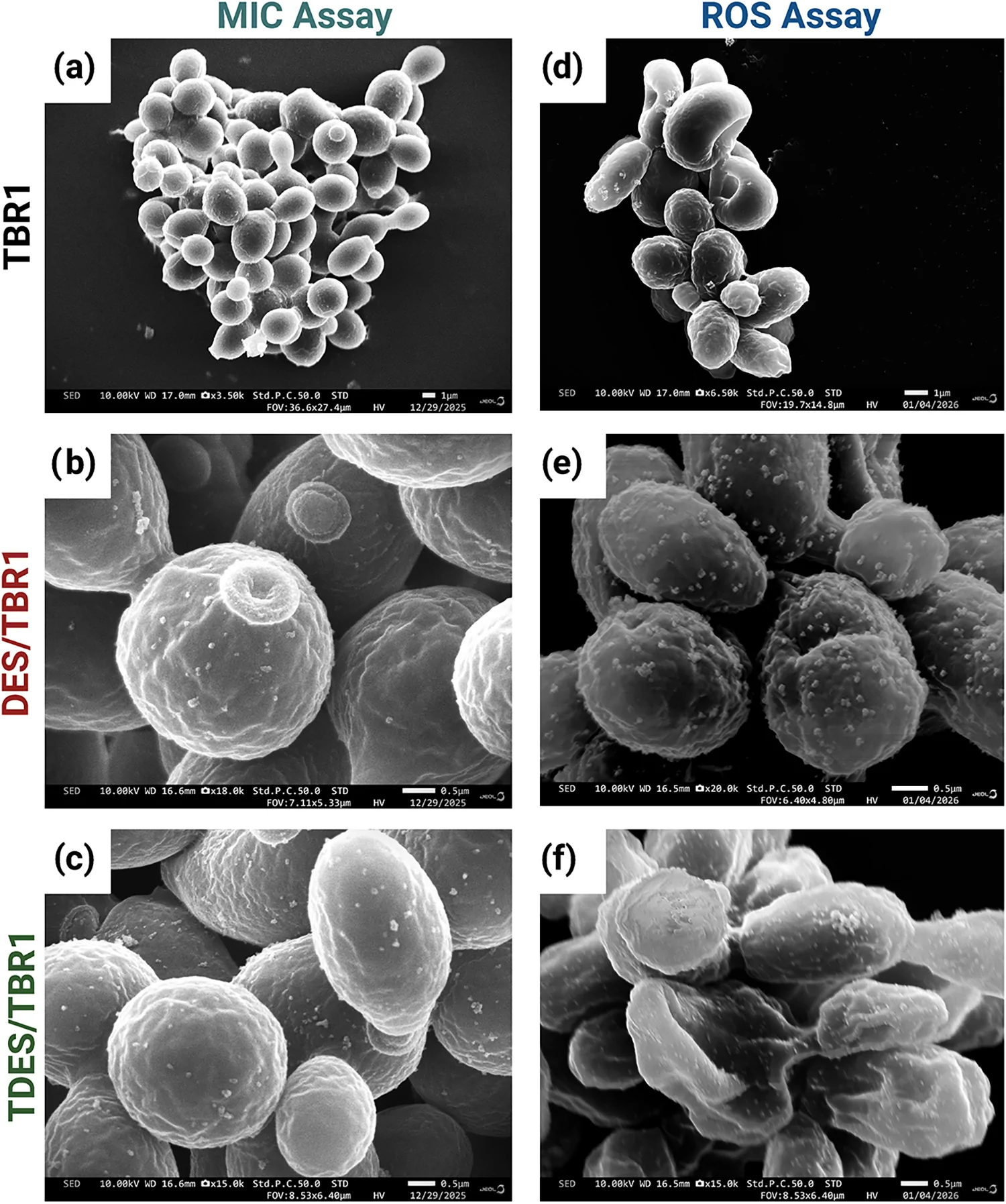
SEM images illustrate the morphological response of TBR1 (*Saccharomyces cerevisiae*) (a, d) following exposure to DES (b, e) and TDES (c,f) under conditions corresponding to MIC and ROS assays.

In contrast, the treated samples (Figure 3(b,c,e,f)) show subtle but noticeable surface changes. Although the overall shape of the cells remains largely intact and no clear rupture or lysis is observed, the cell surfaces appear rougher and are covered with tiny dotlike structures. An important assumption, supported by previous studies on *S. cerevisiae*, is that antifungal activity does not always involve physical destruction of the cell wall.^27,29^ Instead, many antifungal agents act by interacting with the cell surface, particularly the cell membrane and wall, leading to functional disruption rather than immediate structural collapse.^30^ On the basis of this, the tiny dots observed on the cell surface in the SEM images are assumed to represent deposited DES and TDES components triggering localized interactions between the solvent and the cell envelope. These interactions may not visibly break the cell but can disturb the normal organization and function of the membrane.

These observations are important because the yeast cell membrane plays a critical role in regulating what enters and leaves the cell. If DES or TDES components accumulate on the surface, then they may create localized stress points. As a result, this altered permeability is a known mechanism in *S. cerevisiae* stress-response studies and often precedes internal damage. Once these compounds are recognized by the cell, they can trigger the production of ROS. It is well-established in yeast research that elevated ROS levels can damage essential cellular components, including proteins, lipids, and DNA.^31^ Importantly, ROS-induced damage does not require a visible cell rupture.

Instead, it leads to oxidative stress, which can activate apoptosis-like pathways in the yeast cells. In this process, the cell essentially undergoes a controlled form of death while maintaining an intact outer structure. This helps explain the earlier biological results, where strong inhibition of cell growth was observed, even though SEM images do not show obvious cell breakage.

The key assumption here is that cell death occurs internally rather than through mechanical destruction. Additionally, some treated cells, particularly in images from Figure 3(f), appear to be slightly shriveled or irregular. This can be interpreted as early signs of cellular stress, such as loss of membrane integrity, dehydration, or changes in internal pressure, all of which have been reported in ROS-related damage in *S. cerevisiae*. These subtle morphological changes further support the idea that the cells undergo stress-induced damage.

Herein, the SEM images from cells extracted from MIC and ROS assays suggest that DES and TDES exert antifungal effects not by breaking the cells open but by interacting with the cell surface, altering membrane function, and inducing internal oxidative stress, ultimately leading to cell death.

### 3.3 Disruption of the Fungal Cell Wall Analysis

To further elucidate the molecular basis of the observed antifungal activity, ^1^H NMR spectroscopy (Figure 4) was employed to probe alterations in the intracellular biochemical landscape of TBR1 cells following exposure to DES and TDES. The NMR spectrum (Figure 4(a)) corresponds to untreated fungal cells (control), Figure 4(b) corresponds to cells treated with DES, and Figure 4(c) shows fungal cells treated with tannic acid-based DES. In Figure 4(b), several peaks appear that are absent in the control, including a prominent resonance at δ 3.20 ppm corresponding to the trimethylammonium group of choline, signals between δ 3.5 and δ 4.2 ppm assigned to the choline backbone, and aromatic peaks at δ 6.8–7.8 ppm attributed to imidazole.^5,8,32^

**Figure 4:**
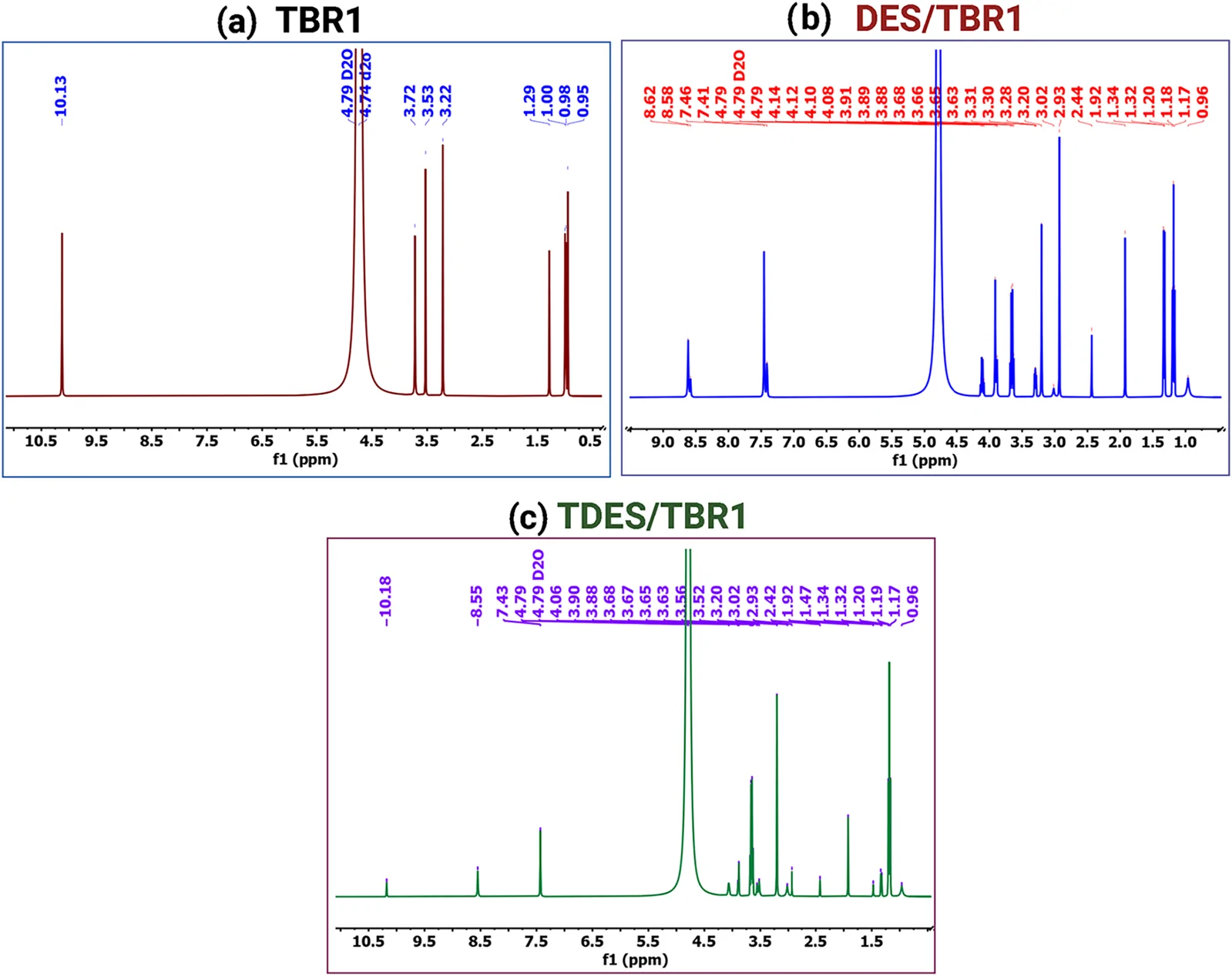
^1^H NMR spectra show how control (a), DES/TBR1 (b), and TDES/TBR1 (c) induced alterations in lipid, carbohydrate, and protein signals, indicating membrane disruption and intracellular biochemical stress.

In contrast, signals characteristic of fungal cells^33^ in Figure 4(a), particularly those in the carbohydrate region (δ 3.2–5.5 ppm) and anomeric region (δ 4.5–5.3 ppm), are not present in cells treated with DES, reflecting the absence of biological material. Slight shifts in DES peaks relative to standard references indicate hydrogen-bonding interactions within the eutectic system. In the TDES-treated sample (Figure 4(c)), several peaks present in the control undergo noticeable chemical shift changes. The anomeric proton signals around δ 4.5–5.3 ppm shift become not defined, indicating alterations in the chemical environment of cell wall polysaccharides such as glucan and chitin due to NMR solvent used.

Peaks in the lipid region (δ 0.9–1.4 ppm) also exhibit small but consistent shifts, suggesting changes in membrane organization. Additionally, peaks observed in DES-treated cells, including the choline signal at δ 3.20 ppm and aromatic signals between δ 6.8–7.8 ppm, reappear in TDES-treated cells, confirming the presence of eutectic components within or associated with the fungal cells.

Importantly, these TDES-related peaks show slight chemical shift differences compared to DES-treated cells, indicating that they are interacting with cellular components rather than existing in their original solvent environment. Additional resonances appearing in regions such as δ 1.2–1.5 ppm and δ 3.3–3.8 ppm suggest redistribution of small metabolites within the system.

These observations collectively indicate that the deep eutectic system successfully affects the fungal cells and alters the chemical environments of polysaccharides, lipids, and proteins. The chemical shift changes in the δ 3.2–5.5 ppm region support disruption of hydrogen bonding in the cell wall, while shifts in the δ 0.9–1.4 ppm region indicate membrane perturbation. Changes in the aromatic region ( δ 6.8–7.8 ppm) suggest interactions involving tannic acid and protein-associated groups. Thus, NMR shows molecular-level alterations supporting a mechanism involving cell wall disruption, membrane destabilization, and protein interaction, leading to growth inhibition and cell death.^34,35^

### 3.4 Inhibiting Fungal Cell Growth Analysis

The disk diffusion images (Figure 5(a–c)) and quantitative data (Table 2) collectively demonstrate the antifungal performance of individual components, DES/TDES systems, and their corresponding eutectogels against TBR1 (*Saccharomyces cerevisiae*). In Figure 5(a), the individual components show distinct behaviors that are confirmed by Table 2. Choline chloride (Figure 5a­(i)) and tannic acid (Figure 5a­(iii)) exhibit no detectable antifungal activity (0 mm ZOI), which is consistent with the absence of visible inhibition zones. In contrast, imidazole (Figure 5a­(ii)) produces a pronounced zone of inhibition (43.67 ± 2.65 mm), indicating that it is the primary active antifungal component within the system. This establishes imidazole as the key contributor to the observed antimicrobial effect. Figure 5(b) shows the activity of the formulated systems. While the control YPD media used to grow fungal cells show no zone of inhibition (Figure 5b­(i)), both DES (ChCl/Imi) (Figure 5b­(ii)) and TDES (ChCl/Imi/TA) (Figure 5b­(iii)) produce clear and well-defined zones of inhibition, which are quantitatively supported by Table 2 (45.11 ± 1.16 mm for DES and 42.44 ± 1.82 mm for TDES). The slightly larger ZOI observed for DES suggests that the addition of tannic acid in TDES does not enhance antifungal activity and may slightly reduce diffusion or availability of the active component. Nonetheless, both systems retain strong antifungal efficacy, indicating that the eutectic formulation preserves the activity of imidazole.

**Table 2:** Antifungal Activity of DES Components, Solutions, and after Being Embedded into Bacterial Nanocellulose^a^

samples	zone of inhibition (mm)
Choline Chloride (ChCl)	0
Imidazole (Imi)	43.67 ± 2.65
Tannic Acid (TA)	0
YPD media	0
Choline Chloride/Imidazole (DES)	45.11 ± 1.16
Choline Chloride/Imidazole/Tannic Acid (TDES)	42.44 ± 1.82
BNC	0
BNC_Eutectogel(Eu)/DES	36.50 ± 2.01
BNC_Eutectogel(Eu)/TDES	32.75 ± 1.26

a Data reported as mean ± standard deviation calculated from three measurements in triplicate per sample, *n* = 3.

**Figure 5:**
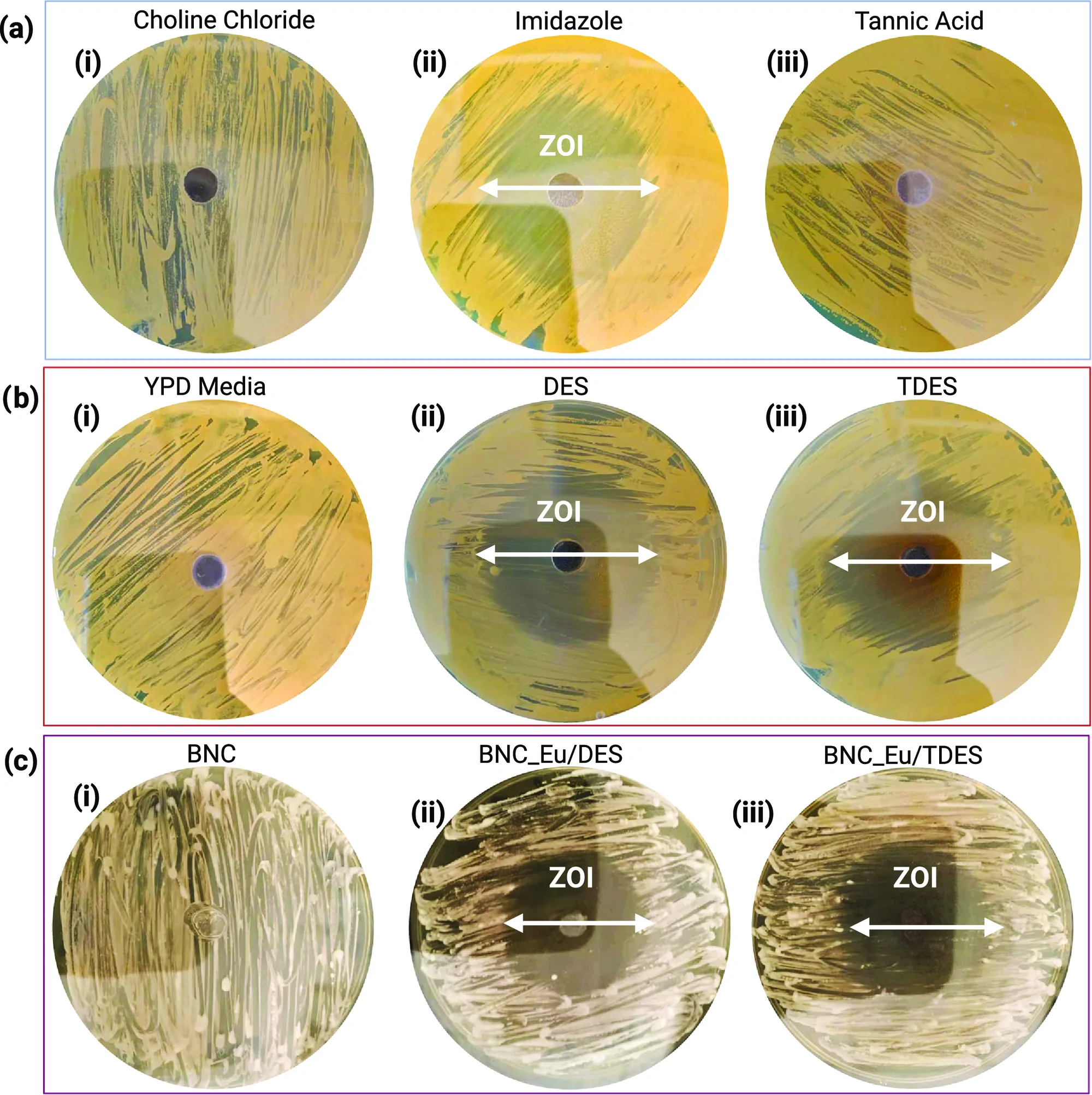
Disk diffusion assays (a–c) showing the zone of inhibition (ZOI) with the most pronounced antifungal effect.

In Figure 5(c), the incorporation of DES and TDES into BNC results in functional eutectogels that maintain antimicrobial activity, as evidenced by visible ZOIs. However, the inhibition zones are reduced compared to the liquid systems, with values of 36.50 ± 2.01 mm for BNC_Eu/DES (Figure 5c­(ii)) and 32.75 ± 1.26 mm for BNC_Eu/TDES (Figure 5c­(iii)). This reduction is expected and can be attributed to the restricted diffusion of active components within the nanocellulose matrix. Importantly, the control BNC shows no inhibition (0 mm) (Figure 5c­(i)), confirming that the antimicrobial effect arises solely from the embedded DES/TDES.

The combined results indicate the following observations: the antifungal activity is primarily driven by imidazole, DES and TDES formulations effectively retain this activity, and embedding into BNC produces a sustained-release system with slightly reduced but still significant antifungal performance.^35,36^ These findings support the successful development of DES/TDES-based eutectogels as functional antimicrobial materials, where activity is preserved while enabling structural integration into a biocompatible matrix.

Although the disk diffusion assay revealed similar inhibition zones between pure imidazole and the deep eutectic systems, this method predominantly reflects passive diffusion behavior through the agar matrix and does not fully capture the intracellular or mechanistic antifungal responses. In the microdilution assays and intracellular ROS measurements, both demonstrated that the deep eutectic systems triggered rapid cell growth inhibition and oxidative stress amplification that was not reproduced by the isolated aqueous components. These findings indicate that the antifungal advantage of deep eutectic formulation is primarily driven, yet does not originate solely from the intrinsic activity of imidazole itself, but from the supramolecular organization of the DES/TDES network during aqueous dilution. This distinction highlights the added value of the deep eutectic systems as a cooperative antifungal delivery solution capable of amplifying cellular stress responses and improving antifungal efficiency beyond what is achievable using conventional aqueous solutions alone.

Beyond the enhanced biological efficacy of the preassembled eutectic matrix, incorporating the DES/TDES system into a bacterial nanocellulose (BNC) eutectogel confers important physicochemical and engineering advantages compared with a conventional BNC hydrogel containing freely dissolved imidazole. In a standard hydrogel system, the water-soluble imidazole remains highly mobile within the bulk aqueous phase, which promotes rapid diffusion, burst release, dehydration, and structural syneresis. In contrast, the eutectic system establishes a supramolecular hydrogen-bonding network among imidazole, choline chloride, and the BNC nanofibers, producing a more stable and coordinated liquid phase confined within the cellulose scaffold.

The synergistic interaction between the BNC network and the eutectic systems is further evidenced by the structural and physicochemical characterization shown in Figure 6. Pure BNC is presented in Figure 6(a), while the BNC-based eutectogels are shown in Figure 6(b) and 6(c). Atomic Force Microscopy (AFM) images (Figure 6(a.1–c.1)) reveal increased surface roughness (Sa) and pit depth (Sv) after incorporation of the DES and TDES systems, indicating nanofiber bundling and reorganization within the cellulose matrix. These observations suggest that the eutectic liquids interact directly with the BNC fibrillar network rather than behaving as passive dissolved additives.

**Figure 6:**
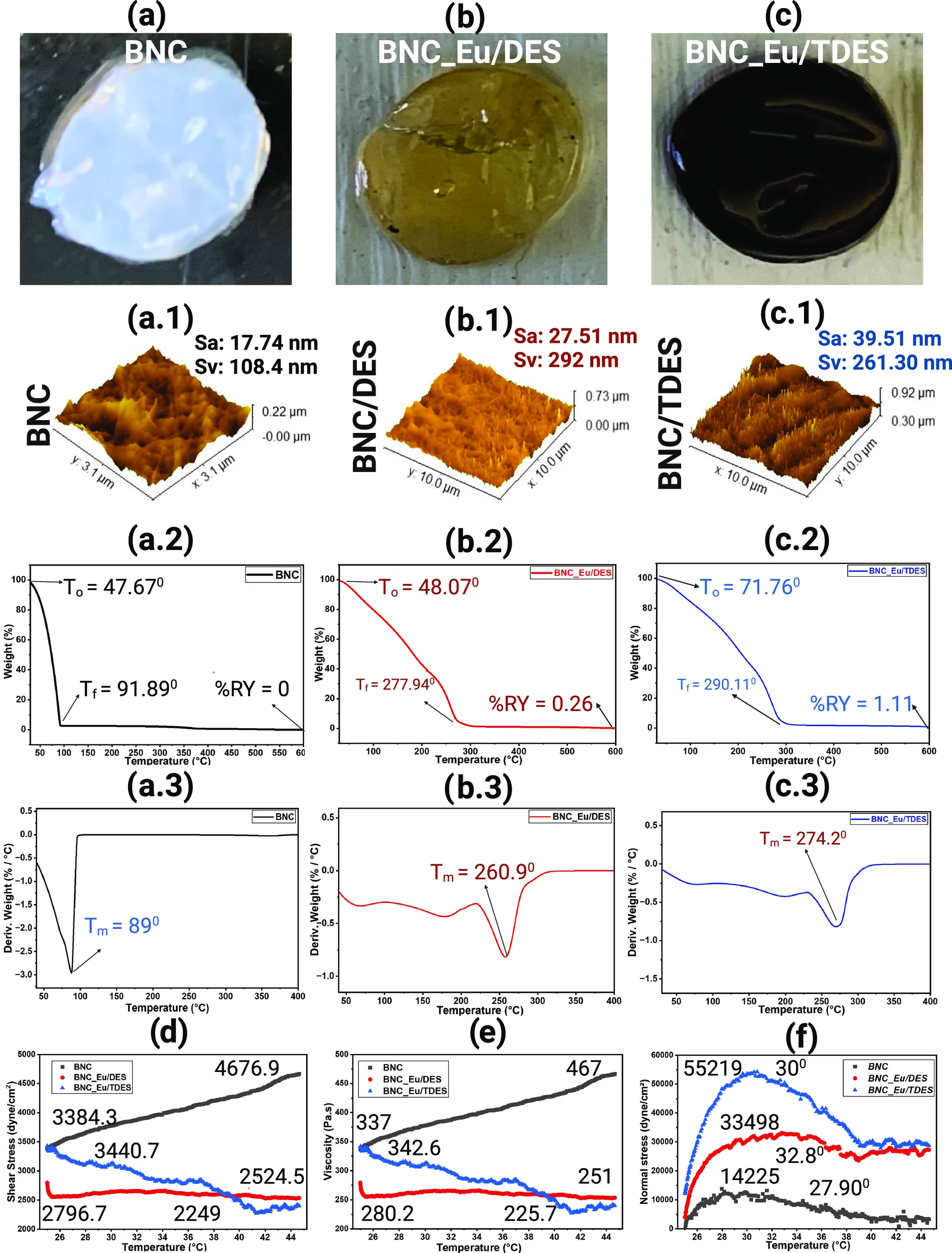
Characterization of BNC-based Eutectogels. (a–c) Digital images; (a.1–c.1) AFM surface morphology; (a.2–c.2) thermal stability and (a.3–c.3) derivative weight using TGA; and mechanical and rheological properties using DMA by measuring (d) shear stress; (e) viscosity; and (f) normal stress.

Thermal analysis further supports this interaction. As shown in Figure 6(a.2–c.2), the eutectogel formulations exhibited substantially improved thermal stability, as reflected by higher final degradation temperatures (T_f_) and residual yields (RY). Among the samples, BNC_Eu/TDES demonstrated the greatest thermal resistance, reaching a final degradation temperature of 290.11 °C with a residual yield of 1.11%. Derivative thermogravimetric analysis (Figure 6(a.3–c.3)) likewise confirmed this stabilization effect, with the major thermal transition shifting markedly from 89.0 °C for pure BNC to 260.9 °C for BNC_Eu/DES and 274.2 °C for BNC_Eu/TDES. Such large increases in thermal stability would not be expected for a conventional hydrogel containing only dissolved imidazole, thereby indicating that eutectic formation contributes directly to matrix stabilization. The rheological properties also demonstrate the beneficial influence of the eutectic systems within the BNC network. While shear stress (Figure 6(d)) and viscosity (Figure 6(e)) decreased following DES incorporation, the normal stress significantly increased (Figure 6(f)), particularly for BNC_Eu/TDES. Specifically, BNC_Eu/TDES achieved 55,219 dyn/cm^2^ at 30 °C, compared with 33,498 dyn/cm^2^ for BNC_Eu/DES at 32.8 °C and only 14,225 dyn/cm^2^ for pure BNC hydrogels at 27.9 °C. This enhancement indicates improved elastic recovery and structural integrity of the eutectogel network despite reduced flow resistance.

These findings demonstrate that the advantage of the bacterial nanocellulose-based eutectogel does not solely arise from the intrinsic bioactivity of imidazole but from the unique supramolecular organization created by the eutectic system within the BNC scaffold. Compared with a conventional hydrogel, the eutectogel exhibits improved nanofiber interaction, enhanced thermal stability, greater structural robustness, and potential for more controlled and sustained drug delivery.

## Conclusion

This study demonstrates that imidazole-based DESs and TDESs exhibit effective antifungal activity against *Saccharomyces cerevisiae* (TBR1) wherein MIC analysis confirmed concentration-dependent growth inhibition, and TDES inducing a stronger ROS response. SEM imaging revealed that treated cells maintained structural integrity without visible rupture but exhibited surface irregularities suggesting a membrane-level disruption. These interactions likely increase membrane permeability, facilitating intracellular stress and ROS accumulation. The elevated ROS levels, combined with reduced cell size and increased cell death, support apoptosis-like processes rather than direct lysis. Metabolite-focused ^1^H NMR analysis further indicated disruption of carbohydrate reserves and central metabolic pathways, reflecting impaired energy homeostasis. This metabolic imbalance, alongside oxidative stress, contributes to reduced cell viability. Importantly, incorporation of DES and TDES into BNC hydrogels produced functional eutectogels that retained antifungal activity with controlled diffusion, highlighting them as promising platforms for antifungal drug-delivery biomaterials system.

## Supplementary Material



## Notes

Sample weight used and final concentration; percent cell viability with standard deviation; MIC assay analysis of TBR1 exposed to choline chloride; MIC assay analysis of TBR1 exposed to Imidazole; MIC assay analysis of TBR1 exposed to tannic acid; and ROS assay analysis of TBR1 exposed to Individual components of DES and TDES (PDF)
