# Supplementary material for: Antifungal and Reactive Oxygen Species Response of Saccharomyces cerevisiae to Deep Eutectic Solvents for Bacterial Nanocellulose-Based Eutectogel
Source: ACS Phys Chem Au. 2026 Jun 25;6(5):1179–91. doi: 10.1021/acsphyschemau.6c00057 (PMC13614122; doi:10.1021/acsphyschemau.6c00057)
Supplement: Supplementary file 1 [file pg6c00057_si_001.pdf]

Supporting Information

Antifungal and Reactive Oxygen Species Response of *Saccharomyces cerevisiae* to Deep Eutectic Solvents for Bacterial Nanocellulose-Based Eutectogel

Maurelio Cabo Jr., Kwaniyah Tuffour, *Dennis LaJeunesse

Department of Nanoscience, Joint School of Nanoscience and Nanoengineering, University of North Carolina Greensboro, Greensboro, North Carolina, 27455, USA

Corresponding Authors: *drlajeun@uncg.edu

Table S1: Sample weight used and final concentration

Samples	Weight (g)	DI Water Added (g)	Final Concentration (mg/mL)
Choline Chloride (ChCl)	8.39	9.53	22.20
Imidazole (Imi)	9.53	8.39	25.20
Tannic Acid (TA)	0.0767	17.92	0.202
Choline Chloride/ Imidazole (DES)	17.92	0	49.20
Choline Chloride/ Imidazole/ Tannic Acid (TDES)	17.9967	0	60.47

Table S2: Percent Cell Viability with Standard Deviation

Sample	Concentration (mg/mL)	% Cell Viability	Standard Deviation (SD)
ChCl	22.20	1.10	0.020
	11.10	3.74	0.111
	5.55	4.43	0.070
	2.78	0.98	0.034
	1.39	1.53	0.049
Imi	25.20	83.01	0.051
	12.60	80.66	0.036
	6.30	77.78	0.018
	3.15	0.469	0.066
	1.58	1.838	0.028
TA	0.202	-2.798	0.053
	0.101	13.34	0.249
	0.051	-0.266	0.045
	0.025	-1.246	0.058
	0.013	2.226	0.056

DES	49.20	87.36	0.029
	24.60	97.41	0.006
	12.30	97.27	0.006
	6.15	92.53	0.017
	3.08	44.68	0.128
TDES	60.47	49.36	0.187
	30.24	77.43	0.083
	15.12	81.04	0.070
	7.56	83.03	0.063
	3.78	30.49	0.257

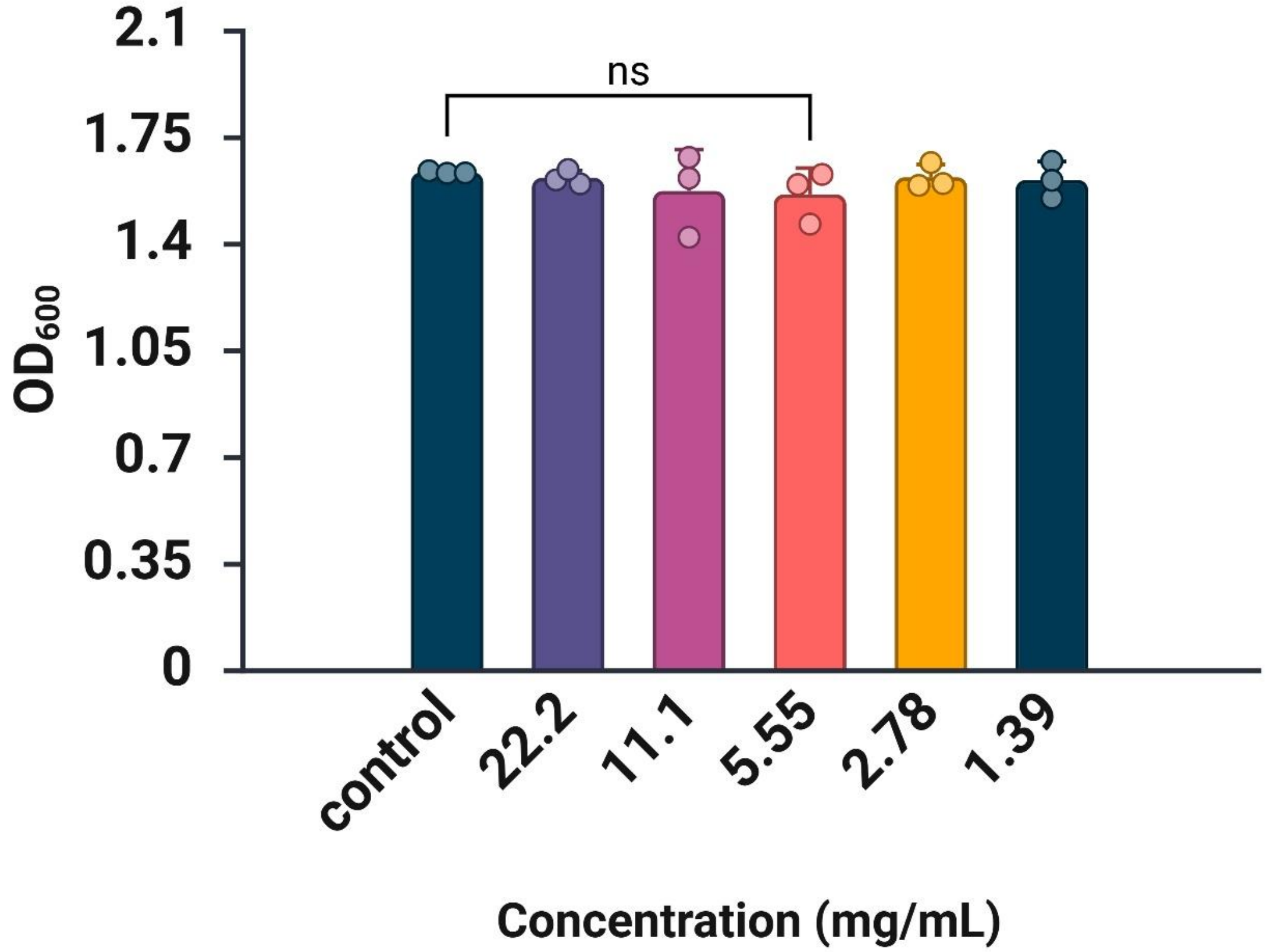

Figure S1: MIC assay analysis of TBR1 exposed to Choline Chloride

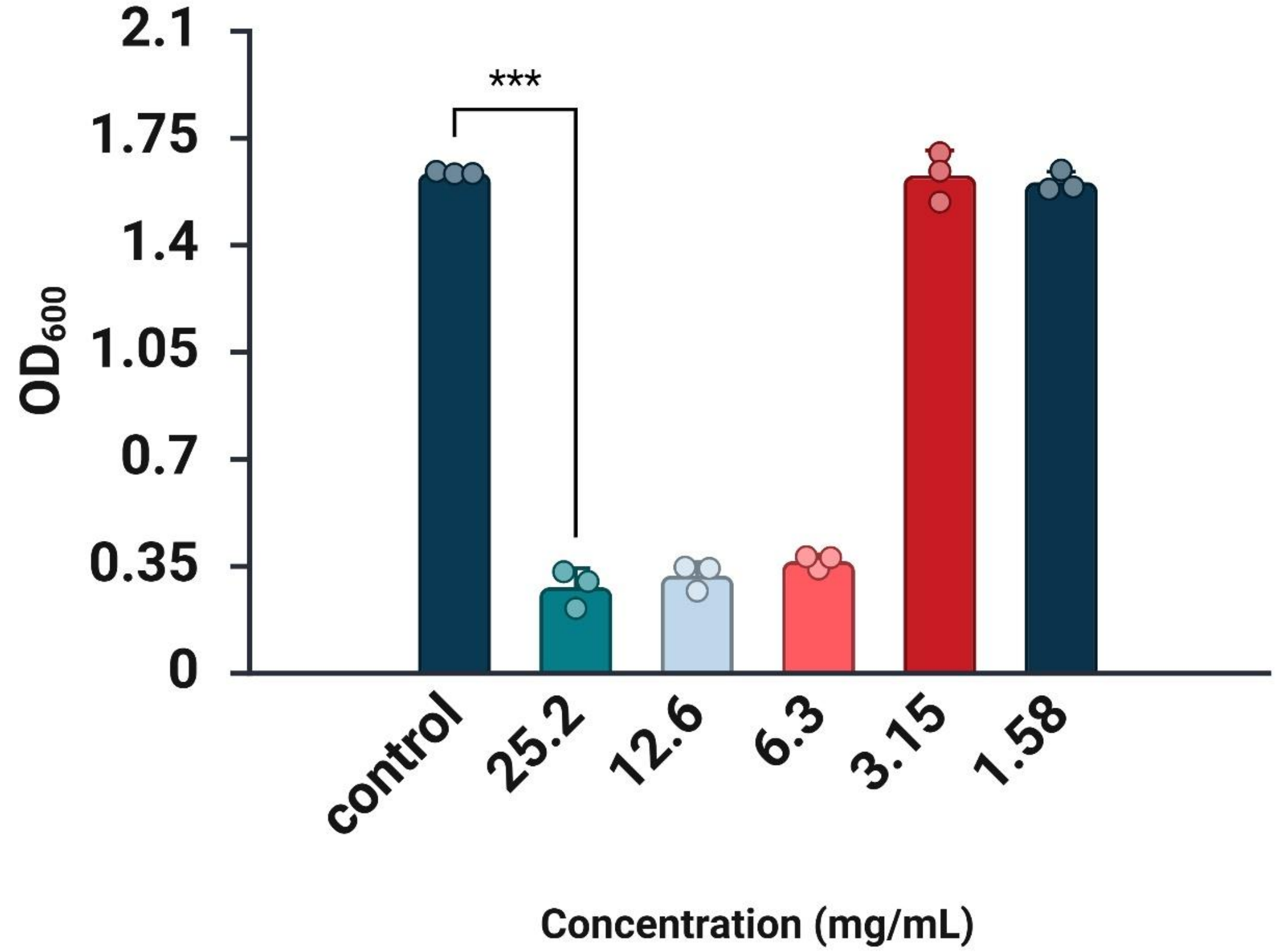

Figure S2: MIC assay analysis of TBR1 exposed to Imidazole

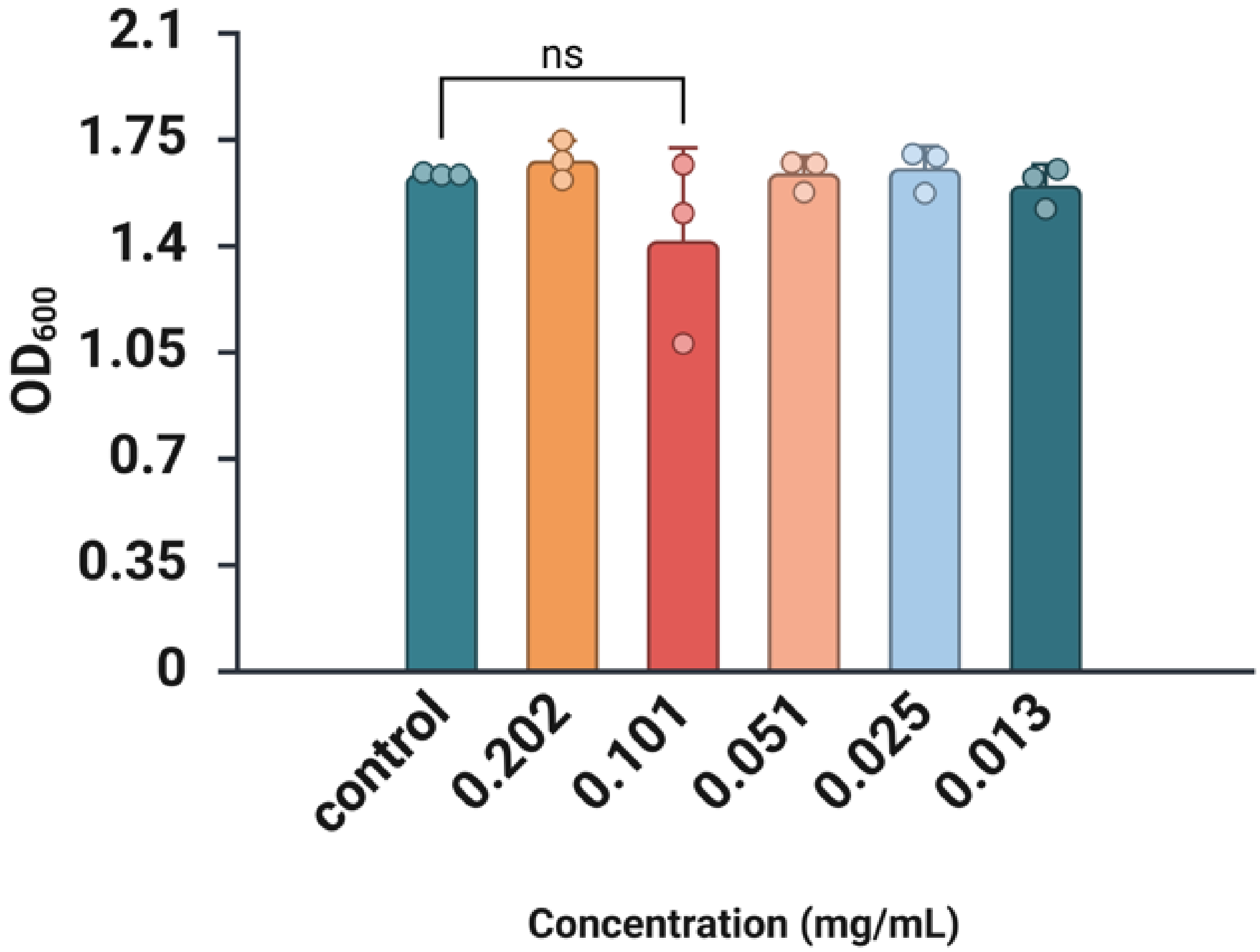

Figure S3: MIC assay analysis of TBR1 exposed to Tannic Acid

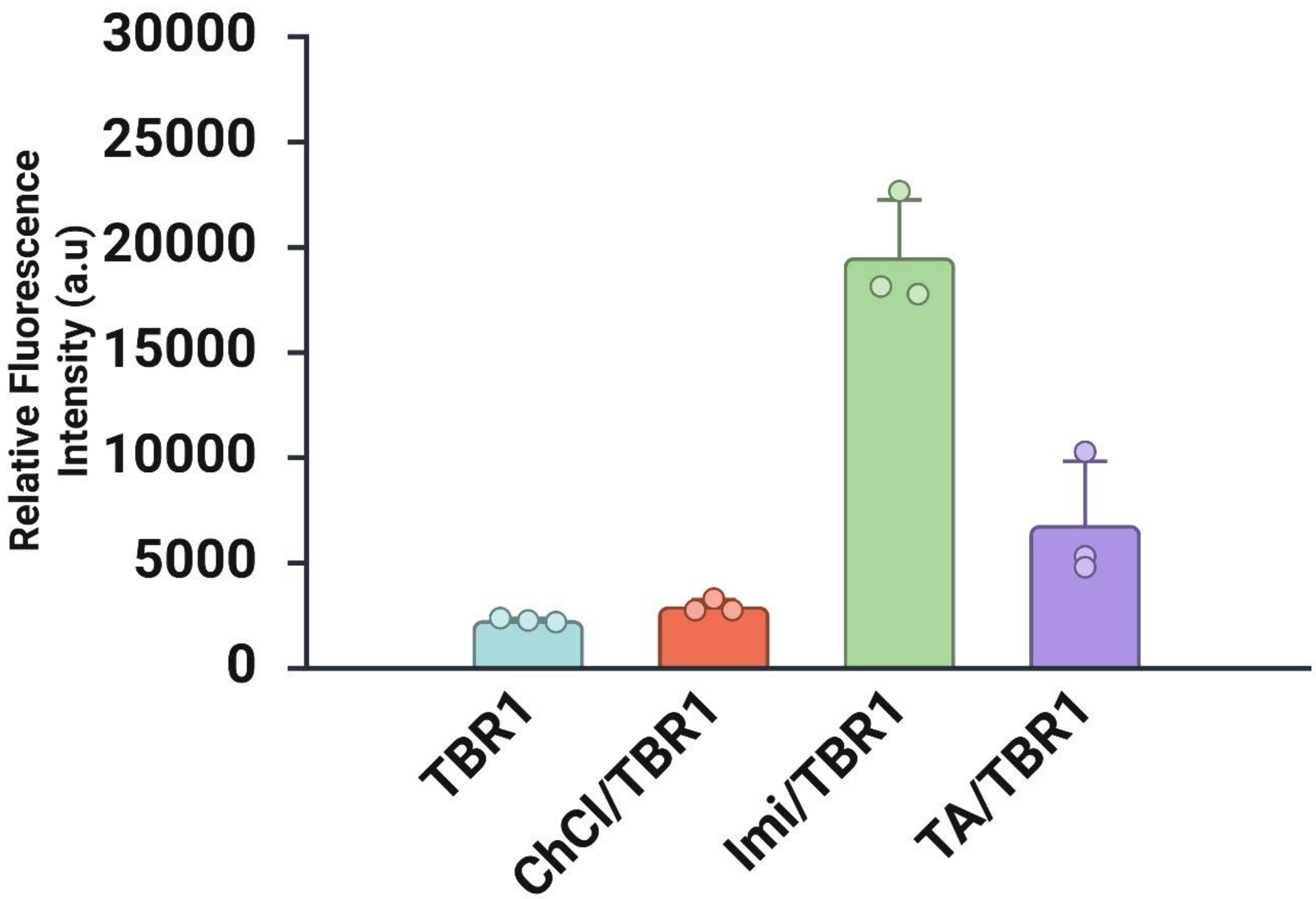

Figure S4: ROS assay analysis of TBR1 exposed to Individual components of DES and TDES
